# Supplementary material for: Deacetylation of β‑Mannans by Two Complementary Carbohydrate Esterases from the Human Gut Microbe Bacteroides cellulosilyticus
Source: Biochemistry. 2026 May 26;65(12):1966–77. doi: 10.1021/acs.biochem.6c00194 (PMC13276840; doi:10.1021/acs.biochem.6c00194)
Supplement: Supplementary file 1 [file bi6c00194_si_001.pdf]

# Supporting information

# Deacetylation of β-Mannans by Two Complementary Carbohydrate Esterases from the Human Gut Microbe *Bacteroides cellulosilyticus*

Lars Jordhøy Lindstad[1], Pascal Michael Mrozek[1], Gordon Jacob Boehlich[1], Shaun Leivers[2], Phillip B. Pope[1,3,4], Sabina Leanti La Rosa[1], Bjørge Westereng[1*]

[1]Faculty of Chemistry, Biotechnology and Food Science, Norwegian University of Life Sciences (NMBU), 1433 Ås, Norway

[2]Nofima AS, Norwegian Institute of Food, Fisheries and Aquaculture Research, 1433 Ås, Norway

[3]Faculty of Biosciences, Norwegian University of Life Sciences (NMBU), 1433 Ås, Norway

[4]Centre for Microbiome Research, School of Biomedical Sciences, Queensland University of Technology (QUT), Translational Research Institute, Woolloongabba 4102, Queensland, Australia

*Corresponding author: Bjørge Westereng, bjorge.westereng@nmbu.no

**Table S1.** Homologs of *Bc*CE25 were identified using pHMMER search against the UniProt database via the EMBL-EBI web server. Reported values include protein entries, taxonomic origin, and E-values. Cutoff values were set to E-value >1e-20 and >30% identity.

| Entry | Organism | E-value |
|---|---|---|
| A0A139KZ99 | Bacteroides intestinalis | 2.1e-164 |
| A0A415IZ39 | Bacteroides sp. AF39-11AC | 2.1e-164 |
| A0A0P0GAI8 | Bacteroides cellulosilyticus | 2.1e-164 |
| A0AAW8VD01 | Bacteroides cellulosilyticus | 2.1e-164 |
| A0A412ICJ7 | Bacteroides cellulosilyticus | 2.0e-163 |
| A0AAW6M6N1 | Bacteroides cellulosilyticus | 2.0e-163 |
| A0A108TGZ9 | Bacteroides cellulosilyticus | 1.3e-161 |
| A0A413H3P4 | Bacteroides stercorirosoris | 6.0e-159 |
| A0A1M6DRX0 | Bacteroides stercorirosoris | 4.1e-158 |
| I8W0W6 | Bacteroides cellulosilyticus | 5.4e-156 |
| A0A9D2CKJ5 | Candidatus Bacteroides pullicola | 5.5e-145 |
| E6STC3 | Bacteroides helcogenes | 5.7e-145 |
| A0A9D2J1X7 | Candidatus Bacteroides merdigallinarum | 1.1e-144 |
| A0A4V3RC77 | Bacteroides muris (ex Afrizal et al. 2022) | 1.2e-144 |
| A0A4Y1VBK2 | Bacteroides uniformis | 2.0e-144 |
| A0AB37MZE3 | Bacteroides sp. AF04-22 | 2.2e-144 |
| A0A3A5SVK7 | Bacteroides sp. AM30-16 | 2.2e-144 |
| A0A8B3E8E1 | Bacteroides sp. AM51-7 | 2.2e-144 |
| E5V844 | Bacteroides sp. 4_1_36 | 2.2e-144 |
| A0A3A5WQ30 | Bacteroides sp. AF34-31BH | 2.2e-144 |

| | | |
|---|---|---|
| A0A3A5Z7D2 | Bacteroides sp. AF25-38AC | 2.2e-144 |
| A0A3A6KVQ3 | Bacteroides sp. AF35-22 | 2.2e-144 |
| A0A3A5R494 | Bacteroides sp. AM44-19 | 2.2e-144 |
| A0A3A6A171 | Bacteroides sp. AF20-13LB | 2.2e-144 |
| A0A3A5RSI4 | Bacteroides sp. AM32-11AC | 2.2e-144 |
| A0A139K8T6 | Bacteroides uniformis | 2.2e-144 |
| A0A285RN65 | Bacteroides sp. AR29 | 2.2e-144 |
| A0AB37MPI2 | Bacteroides sp. AF16-7 | 2.2e-144 |
| A0AB37L9F5 | Bacteroides sp. D20 | 3.0e-144 |
| A0A414BKG6 | Bacteroides uniformis | 3.0e-144 |
| A0A3A5XL82 | Bacteroides sp. AF29-11 | 3.0e-144 |
| A0A3A5RPA5 | Bacteroides sp. AM41-16 | 3.0e-144 |
| A0A414IFJ4 | Bacteroides uniformis | 3.0e-144 |
| A0A9X2NQ02 | Bacteroides muris (ex Fokt et al. 2023) | 4.2e-144 |
| A0A9D2A707 | Candidatus Bacteroides merdipullorum | 5.1e-144 |
| A0AA37JP65 | Bacteroides uniformis | 5.4e-144 |
| A0A4P6RFS1 | Bacteroides sp. A1C1 | 5.7e-144 |
| A0A3E5EPV8 | Bacteroides uniformis | 5.7e-144 |
| A0A412JMT6 | Bacteroides uniformis | 7.8e-144 |
| R9HQL6 | Bacteroides uniformis | 7.8e-144 |
| A0A9X2NX67 | Bacteroides muris (ex Fokt et al. 2023) | 8.0e-144 |
| A0A412BA43 | Bacteroides uniformis | 1.1e-143 |
| A0A3A5P7B2 | Bacteroides sp. AF39-16AC | 1.1e-143 |
| A0A413N8Z6 | Bacteroides uniformis | 2.1e-143 |
| A0A7D6Y2I1 | Bacteroides sp. CACC 737 | 2.1e-143 |

| | | |
|---|---|---|
| A0A3A5YPX2 | Bacteroides sp. AF25-17LB | 2.1e-143 |
| A0A9E2NPQ2 | Candidatus Bacteroides intestinipullorum | 3.8e-143 |
| A0AAW6GJE5 | Bacteroides uniformis | 5.4e-143 |
| A0A5C6HJE8 | Bacteroidaceae bacterium HV4-6-C5C | 1.5e-140 |
| A0A840CXM7 | Bacteroides reticulotermitis | 2.0e-129 |
| A0A1C5QN83 | uncultured Bacteroides sp. | 9.2e-116 |
| A0A1G6G7I5 | Bacteroides ovatus | 5.1e-115 |
| A0A373GHG6 | Bacteroides sp. AM56-10ce | 6.8e-114 |
| A0A3A6MHX4 | Bacteroides sp. OF03-11BH | 6.8e-114 |
| A0A413VHI9 | Bacteroides ovatus | 6.8e-114 |
| A0A414JLS2 | Bacteroides uniformis | 1.9e-113 |
| E5CAV3 | Bacteroides sp. D2 | 2.5e-113 |
| A0A3A5ZTV2 | Bacteroides sp. AF25-38AC | 6.9e-113 |
| A0A173Y0N0 | Bacteroides uniformis | 6.9e-113 |
| A0A8B3ECM4 | Bacteroides sp. AM51-7 | 6.9e-113 |
| A0A3A5WHH0 | Bacteroides sp. AF34-31BH | 6.9e-113 |
| A0A3A5QNT9 | Bacteroides sp. AM44-19 | 6.9e-113 |
| A0A078S4K9 | Bacteroides uniformis | 1.1e-109 |
| A0A3E4XKX0 | Bacteroides uniformis | 1.3e-108 |
| A0A940Q3K7 | Oscillospiraceae bacterium | 8.7e-82 |
| R5L4Z7 | Eubacterium sp. CAG:115 | 1.9e-81 |
| A0A928L150 | Ruminococcus sp. | 1.4e-80 |
| R5H680 | Eubacterium sp. CAG:786 | 2.2e-80 |
| A0A940Q7A3 | Oscillospiraceae bacterium | 2.7e-80 |
| R7KXC3 | Ruminococcus sp. CAG:353 | 3.2e-80 |

| | | |
|---|---|---|
| A0A1C6B2C0 | uncultured Ruminococcus sp. | 3.2e-80 |
| A0A9C7QG62 | Oscillospiraceae bacterium | 3.1e-76 |
| A0A5M5ZLI9 | Phocaeicola dorei | 5.5e-70 |
| A0A4R0N0B4 | Pedobacter hiemivivus | 1.1e-40 |
| A0A1I2BVZ9 | Chitinophaga sp. CF118 | 6.9e-39 |
| A0A927J8Y5 | Dysgonomonas sp. BGC7 | 1.8e-36 |
| A0A9D9HGG9 | Candidatus Cryptobacteroides merdigallinarum | 8.5e-33 |
| A0A9D9HI80 | Candidatus Cryptobacteroides intestinavium | 3.6e-32 |
| A0A8I0VEJ9 | Acinetobacter sp. SK-43 | 4.9e-20 |

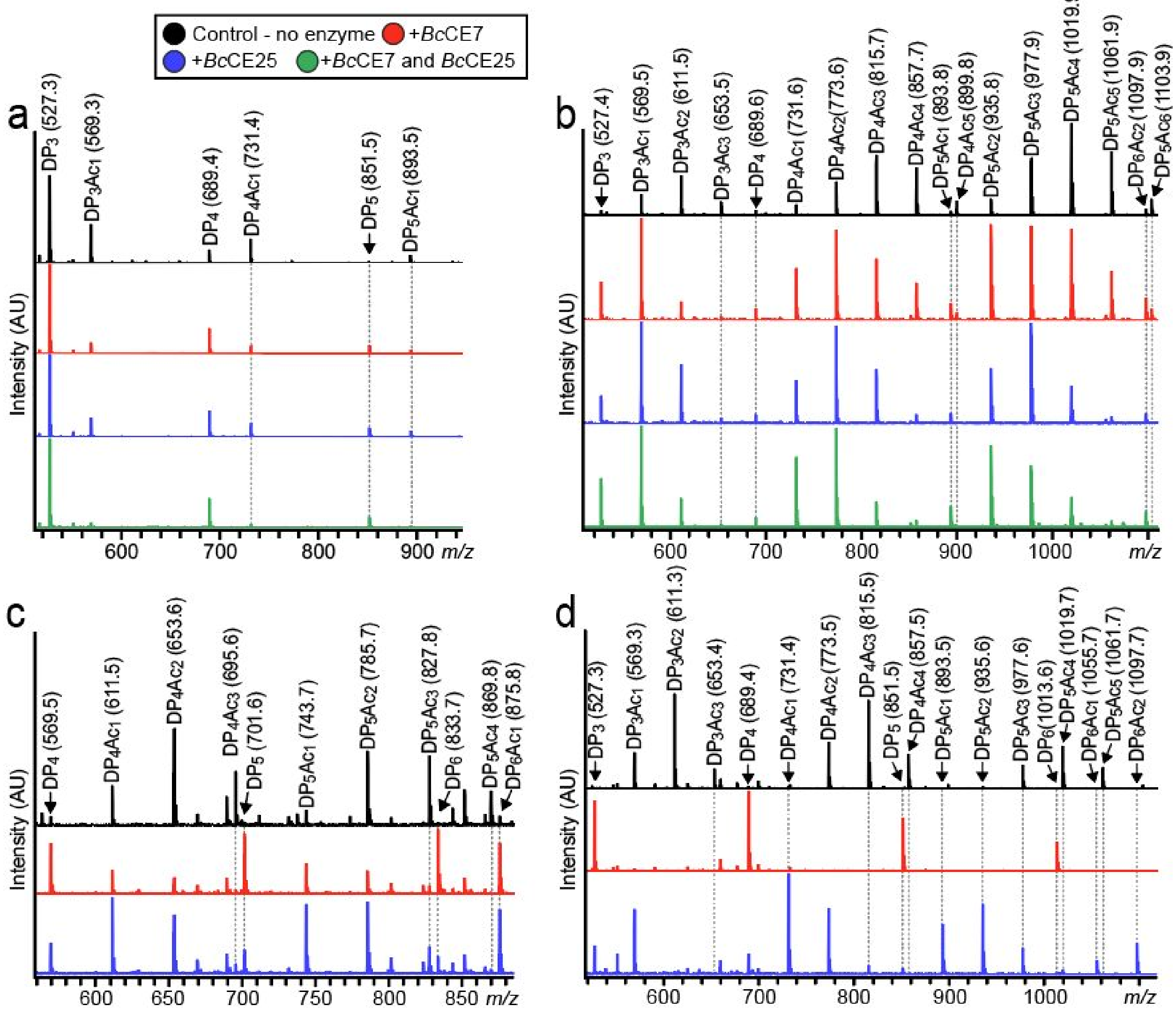


**Figure S1.** MALDI-ToF spectra of enzyme reactions by *Bc*CE7 and *Bc*CE25 on various substrates. Deacetylation of *Ri*GH26-digested KGM (a) and *A. vera* mannan (b). Deacetylation of acetylated xylan (c) and cellulose monoacetate (d). The reactions were carried out with 1 μM enzyme concentration and 0.1 mg/mL substrate in 10 mM sodium phosphate pH 5.9 buffer at 25 ˚C with stirring for 24 h. Abbreviations: Ac, acetyl; DP, degree of polymerization; *m/z*, mass/charge. Grey dotted vertical lines are included to aid interpretation of peaks of closely spaced or identical *m/z* values in overlaid spectra.

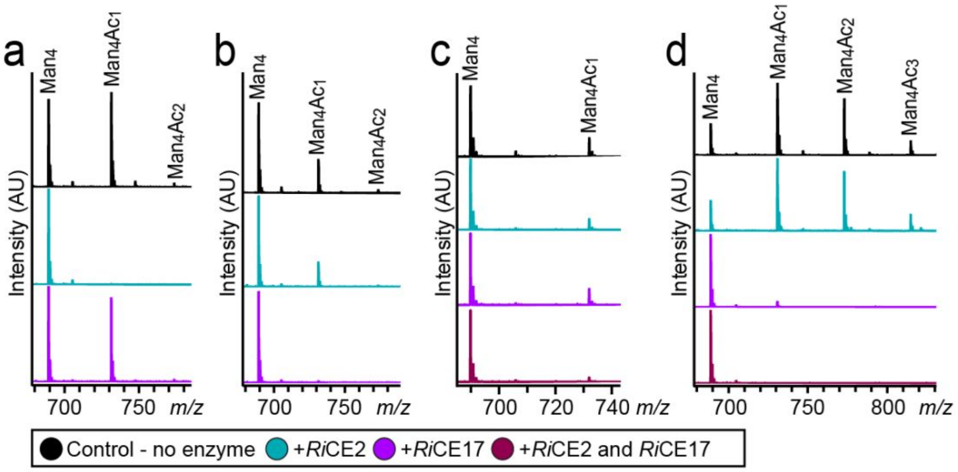


**Figure S2.** Test of *Ri*CE2 and *Ri*CE17 in deacetylation reactions on transacetylated products; Deacetylation of mannotetraose transacetylated with *Ri*CE2 (a) and *Ri*CE17 (b) (to control that no acetyl migration occurred during the production of these substrates), and with *Bc*CE7 (c) and *Bc*CE25 (d). The reactions were carried out with 1 μM enzyme concentration and 0.1 mg/mL substrate in 10 mM sodium phosphate pH 5.9 buffer at 25 ˚C with stirring for 1 h. Abbreviations: Ac, acetyl; Man, mannose; *m/z*, mass/charge.

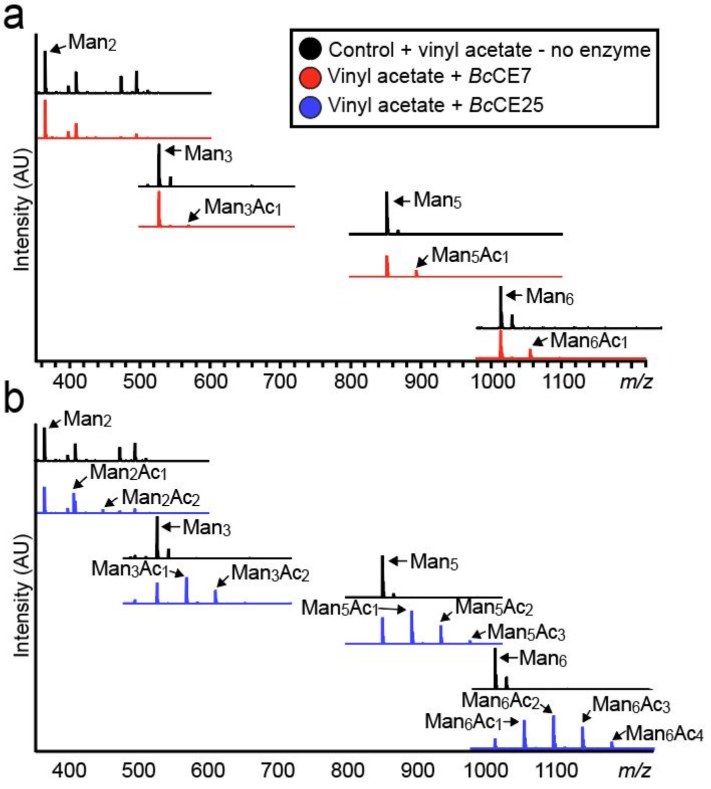

**Figure S3.** a) Transacetylation of $Man_2$, $Man_3$, $Man_5$, and $Man_6$ with *Bc*CE7. No acetyl group was observed for $Man_2$, while mainly one acetylation was added to the larger mannooligosaccharides. b) *Bc*CE25 transacetylated all tested substrates, with multiple acetylations for each. Transesterification reactions were conducted with 1 mg/mL substrate in 10 mM sodium phosphate (pH 5.9) and 200 nM enzyme with vinyl acetate donors added to 50% of the sample volume and run overnight with stirring at 25 ˚C. Abbreviations: Ac, acetyl; Man, mannose; *m/z*, mass/charge.

**NMR Data of acetylated mannotetraose products**

**Table S2**. Peak assignments for acetylated mannotetraose units.

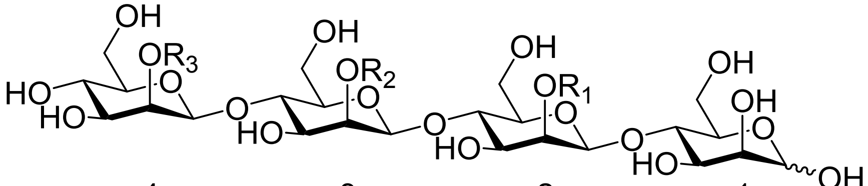


4 3 2 1

| | Mannose-unit | | |
|---|---|---|---|
| | 4<br>$R_3$ = Ac | 3<br>$R_2$ = Ac | 2<br>$R_1$ = Ac |
| 1 H | 4.94 | 4.96 | 4.92 |
| C | 102.1 | 102.1 | 102.1 |
| 2 H | 5.47 | 5.53 | 5.49 |
| C | 75.1 | 74.5 | 74.6 |
| 3 H | 3.86 | 4.02 | 3.97 |
| C | 74.2 | 73.0 | 73.4 |
| 4 H | 3.60 | 3.87 | 3.85 |
| C | 69.9 | 79.5 | 79.5 |
| 5 H | 3.53 | 3.64 | 3.54 |
| C | 78.0 | 78.2 | 77.9 |
| 6 $H_1$ | 3.72 | 3.78 | 3.78 |
| $H_2$ | 3.95 | 3.95 | 3.95 |
| C | 64.0 | 63.5 | 63.5 |

a

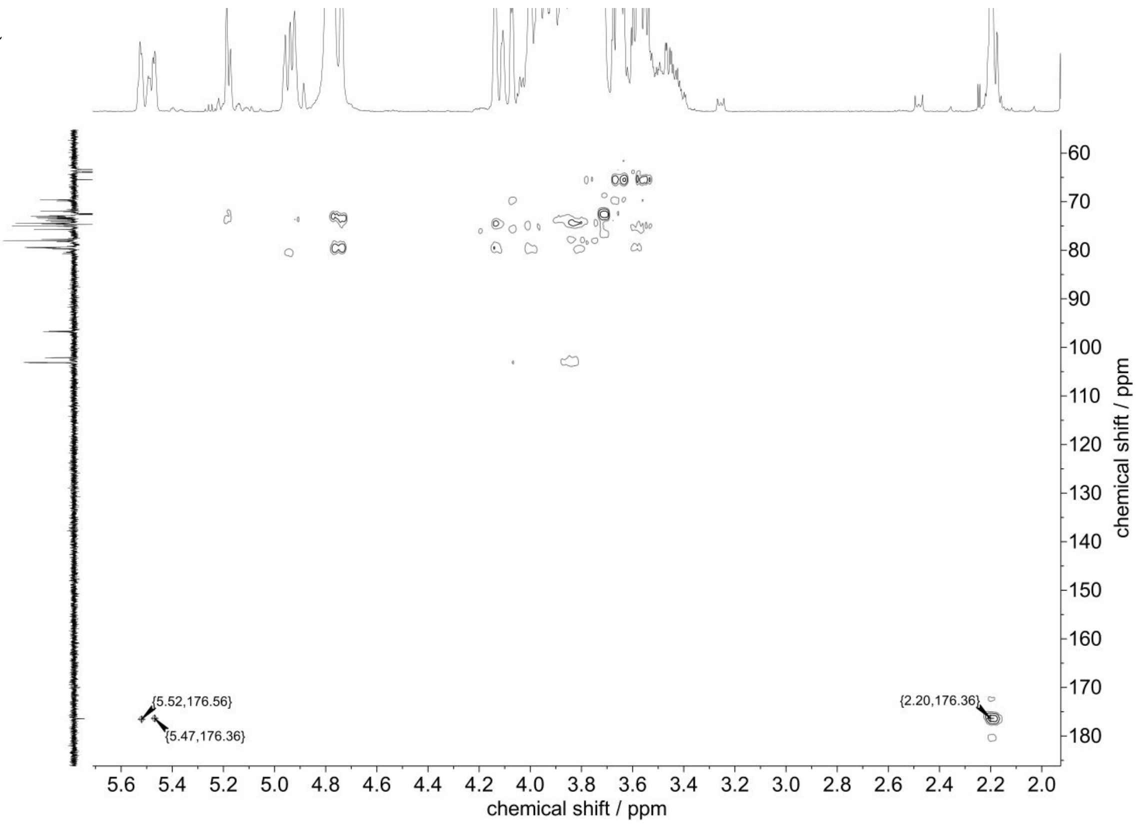
{5.52,176.56}
{5.47,176.36}
{2.20,176.36}
chemical shift / ppm
chemical shift / ppm

b

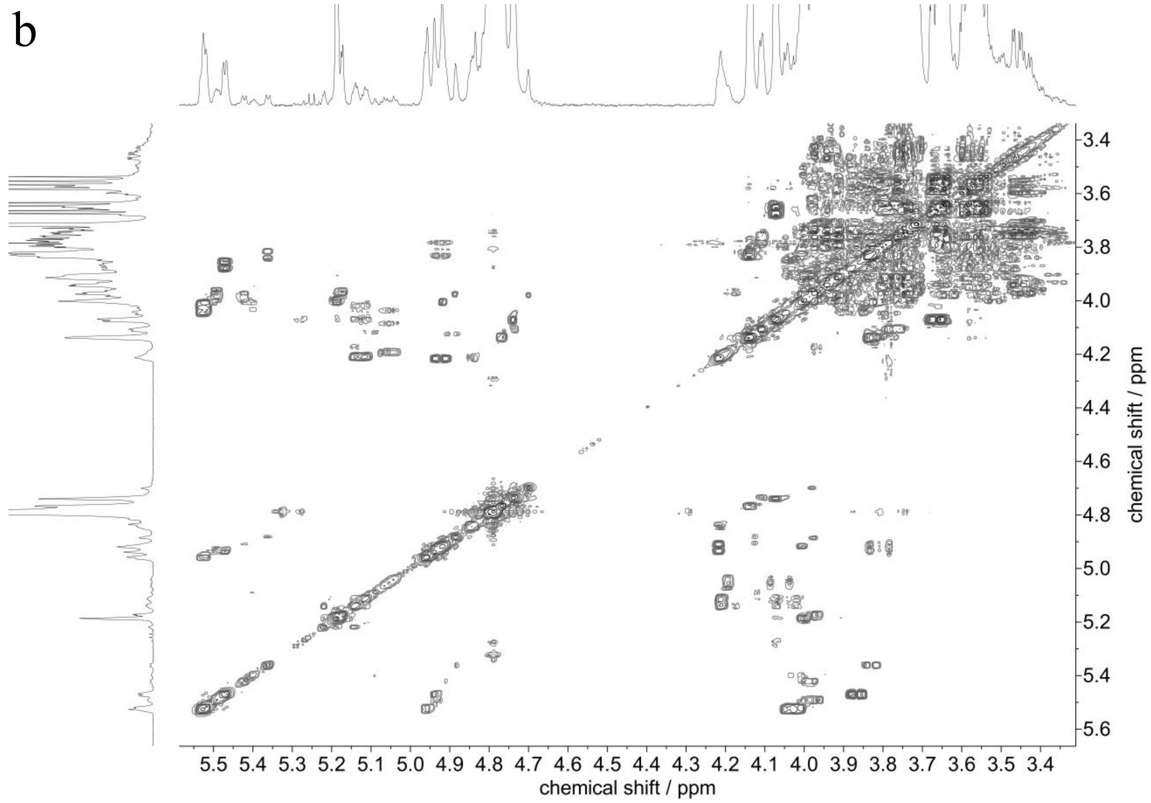
chemical shift / ppm
chemical shift / ppm

c

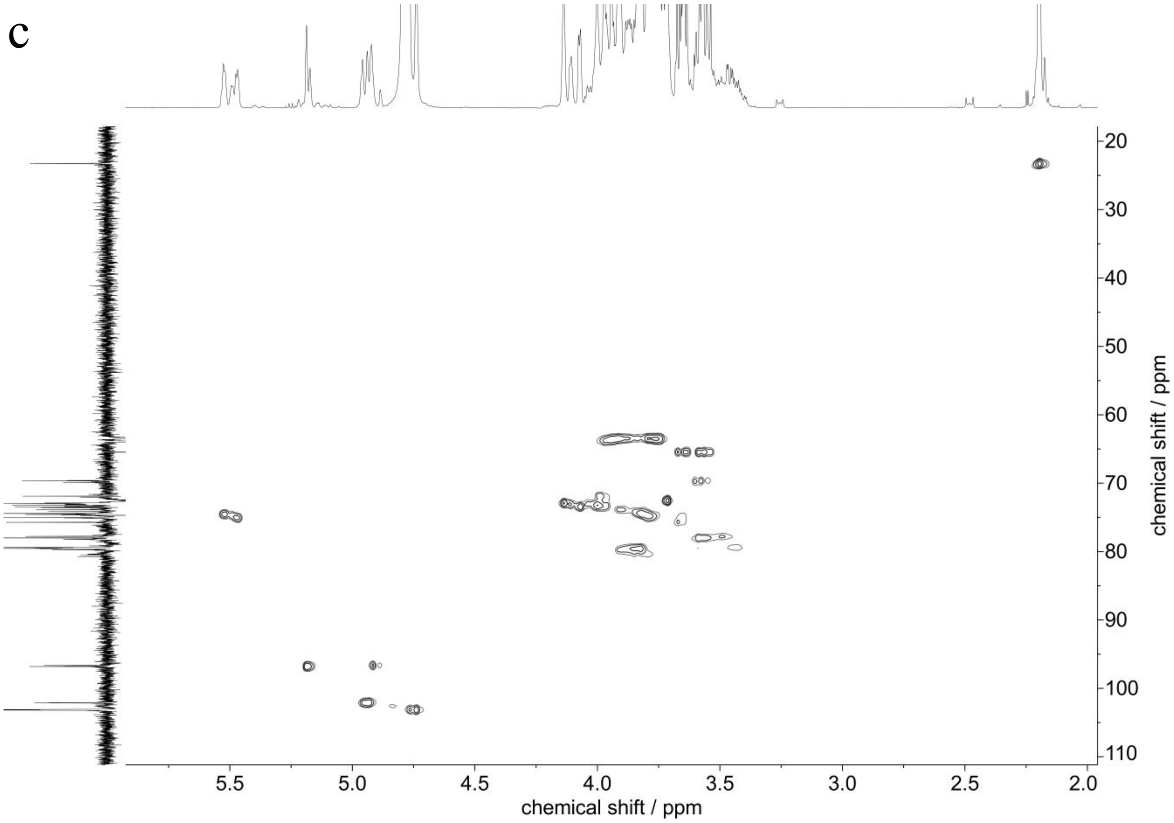

chemical shift / ppm
chemical shift / ppm


d

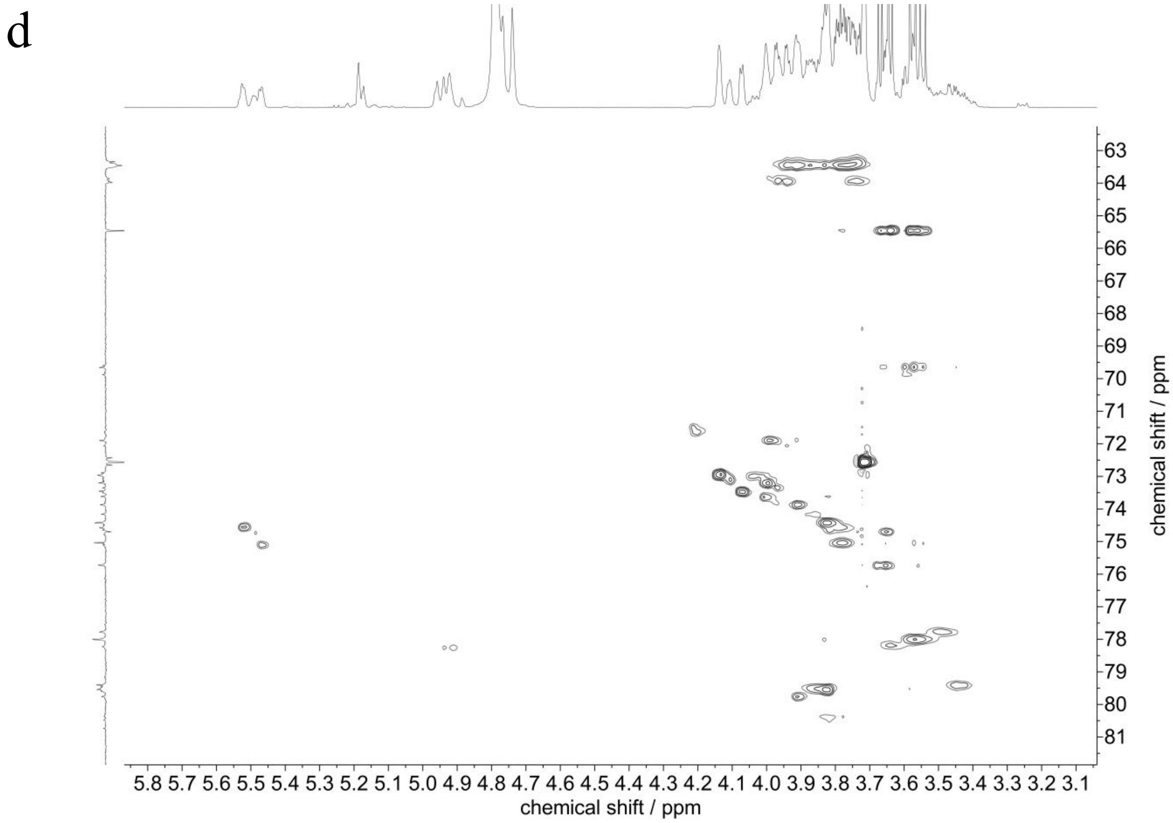

chemical shift / ppm
chemical shift / ppm

e

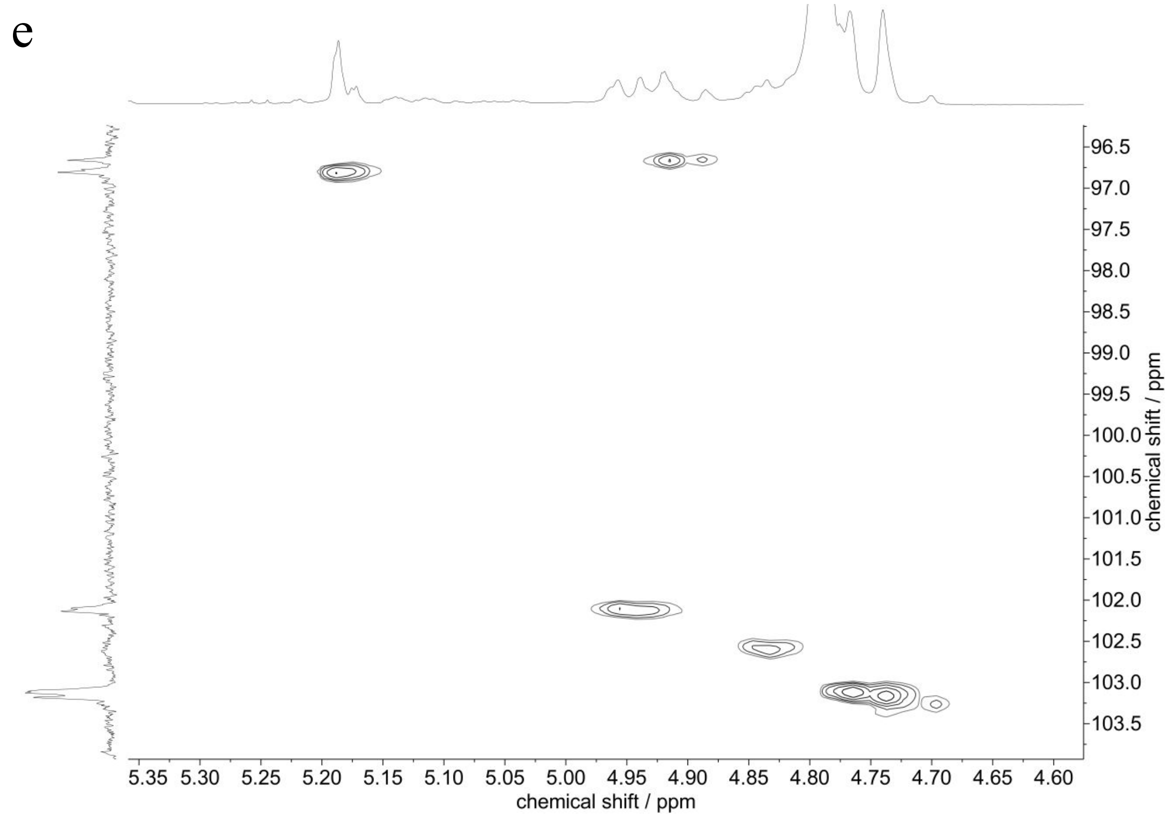

chemical shift / ppm
chemical shift / ppm


f

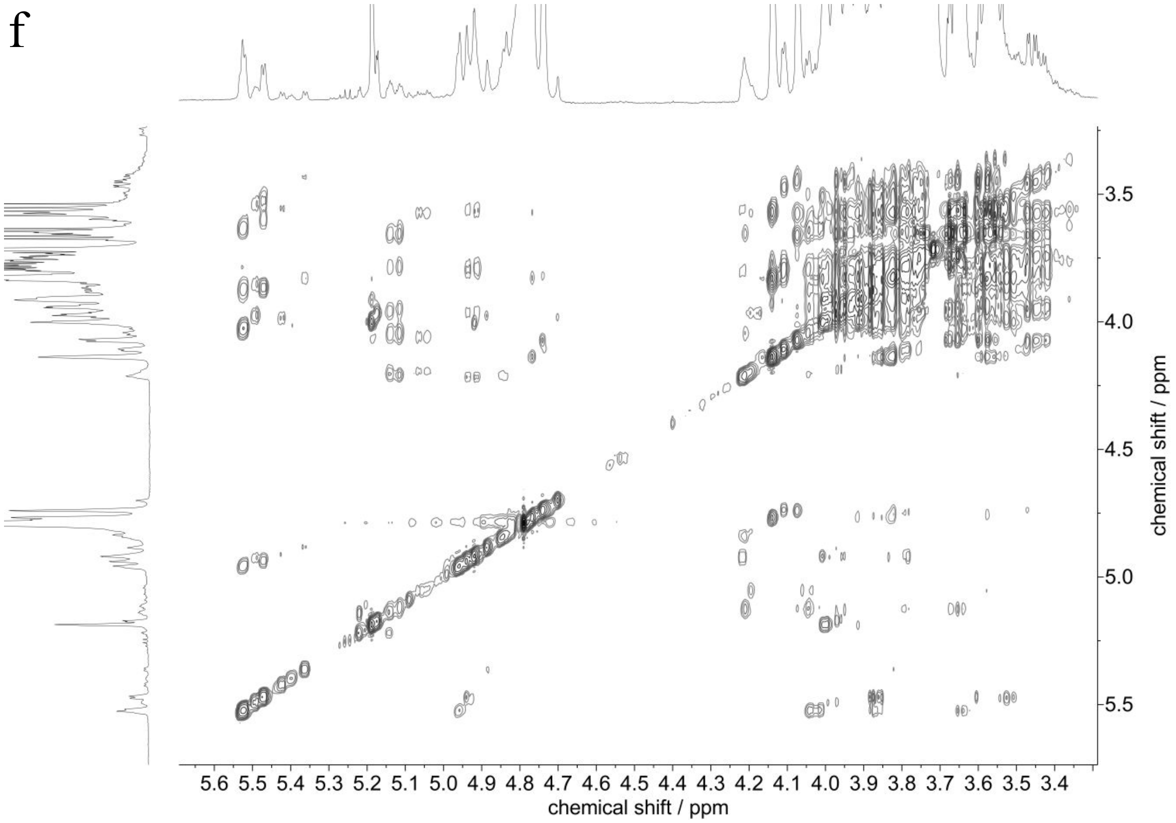

chemical shift / ppm
chemical shift / ppm

g

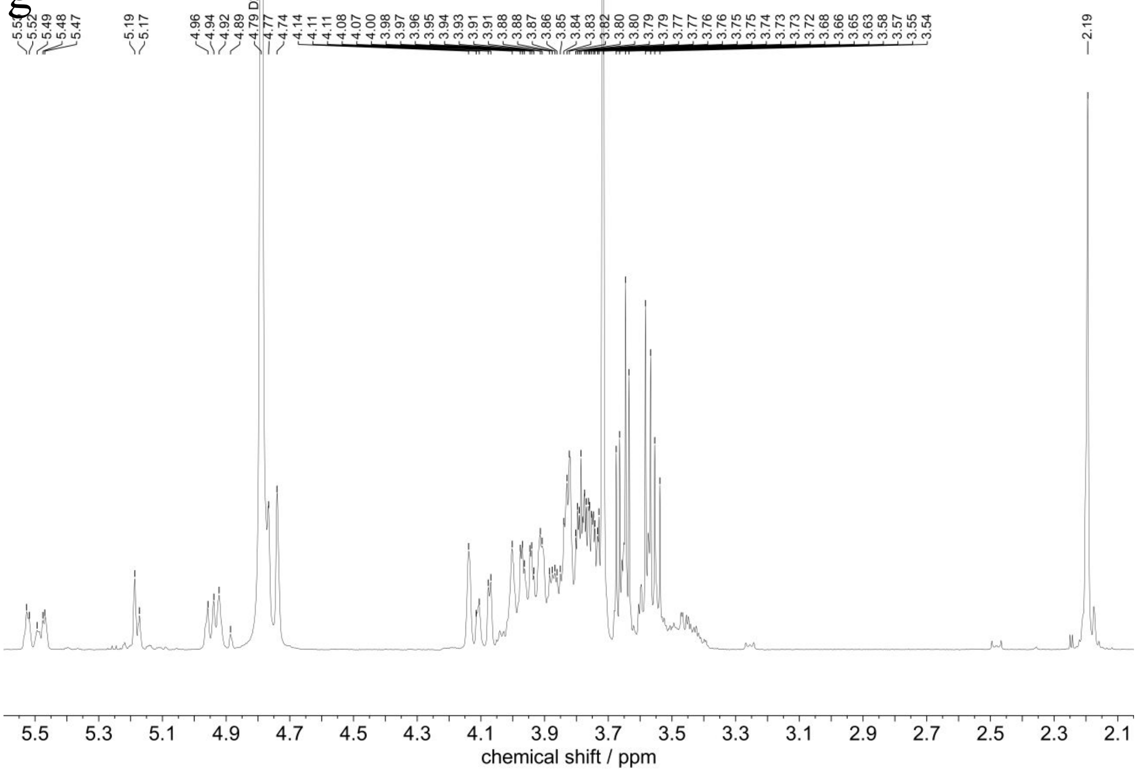

5.53 5.52 5.49 5.48 5.47
5.19 5.17
4.96 4.94 4.92 4.89 4.79 D2O 4.77 4.74
4.14 4.11 4.11 4.08 4.07 4.00 3.98 3.97 3.96 3.95 3.94 3.93 3.91 3.91 3.88 3.88 3.87 3.86 3.85 3.84 3.83 3.82 3.80 3.80 3.79 3.79 3.77 3.77 3.76 3.76 3.75 3.75 3.74 3.73 3.73 3.72 3.68 3.66 3.65 3.63 3.58 3.57 3.55 3.54
2.19
5.5 5.3 5.1 4.9 4.7 4.5 4.3 4.1 3.9 3.7 3.5 3.3 3.1 2.9 2.7 2.5 2.3 2.1
chemical shift / ppm


h

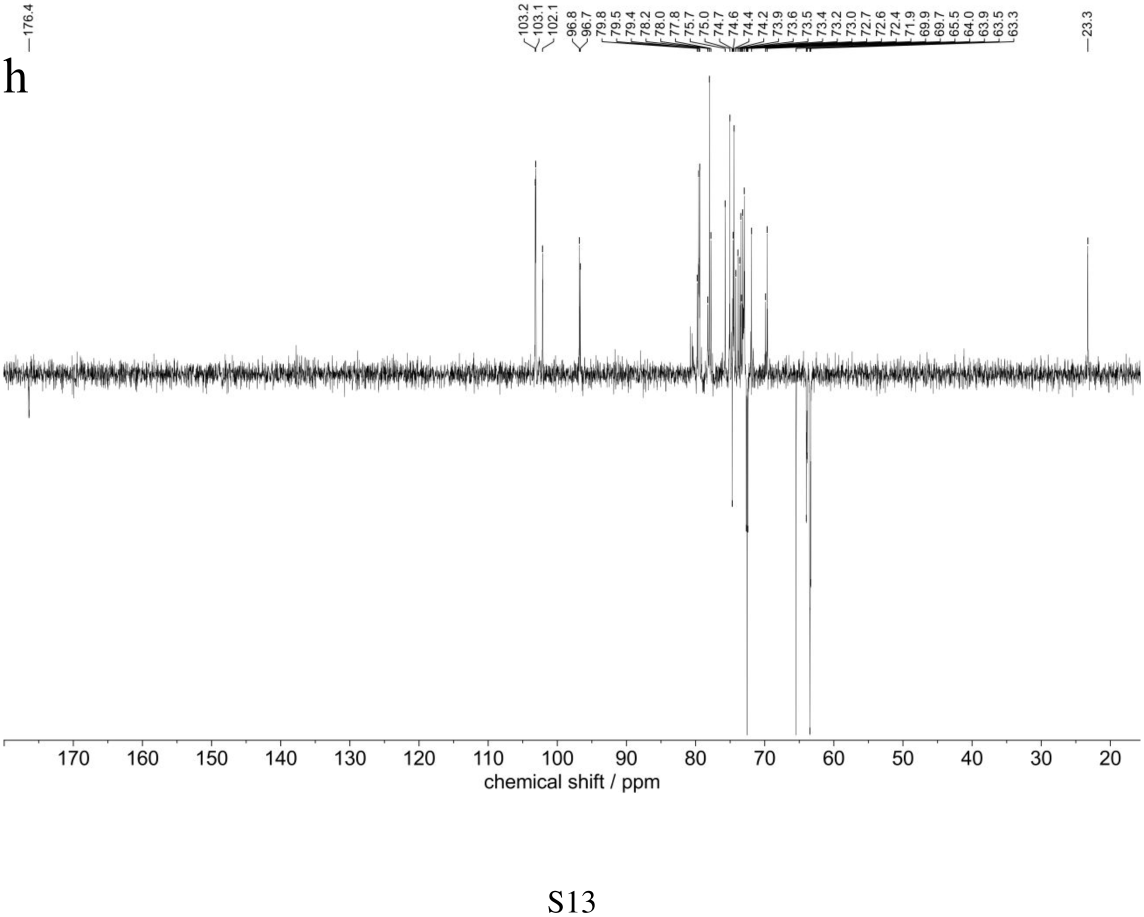

176.4
103.2 103.1 102.1 96.8 96.7
79.8 79.5 79.4 78.2 78.0 77.8 75.7 75.0 74.7 74.6 74.4 74.2 73.9 73.6 73.5 73.4 73.2 73.0 72.7 72.6 72.4 71.9 69.9 69.7 65.5 64.0 63.9 63.5 63.3
23.3
170 160 150 140 130 120 110 100 90 80 70 60 50 40 30 20
chemical shift / ppm

i

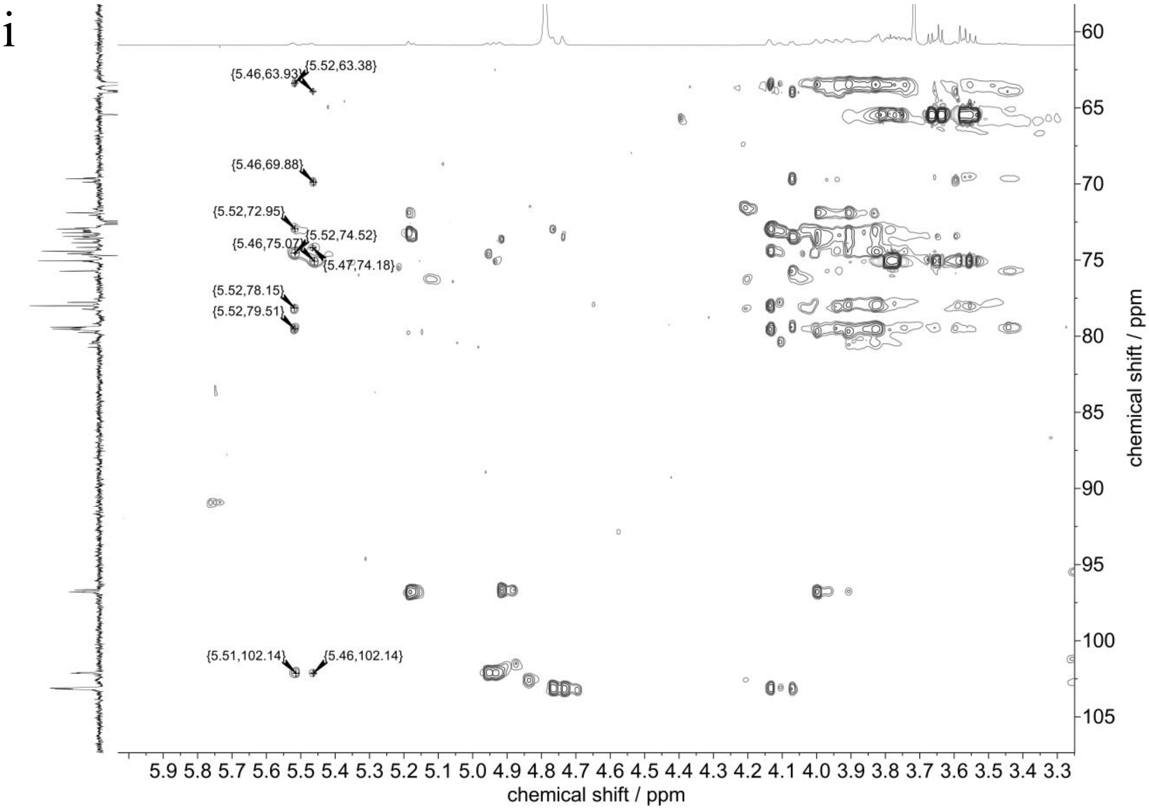
{5.46,63.93}
{5.52,63.38}
{5.46,69.88}
{5.52,72.95}
{5.52,74.52}
{5.46,75.07}
{5.47,74.18}
{5.52,78.15}
{5.52,79.51}
{5.51,102.14}
{5.46,102.14}
chemical shift / ppm
chemical shift / ppm

j

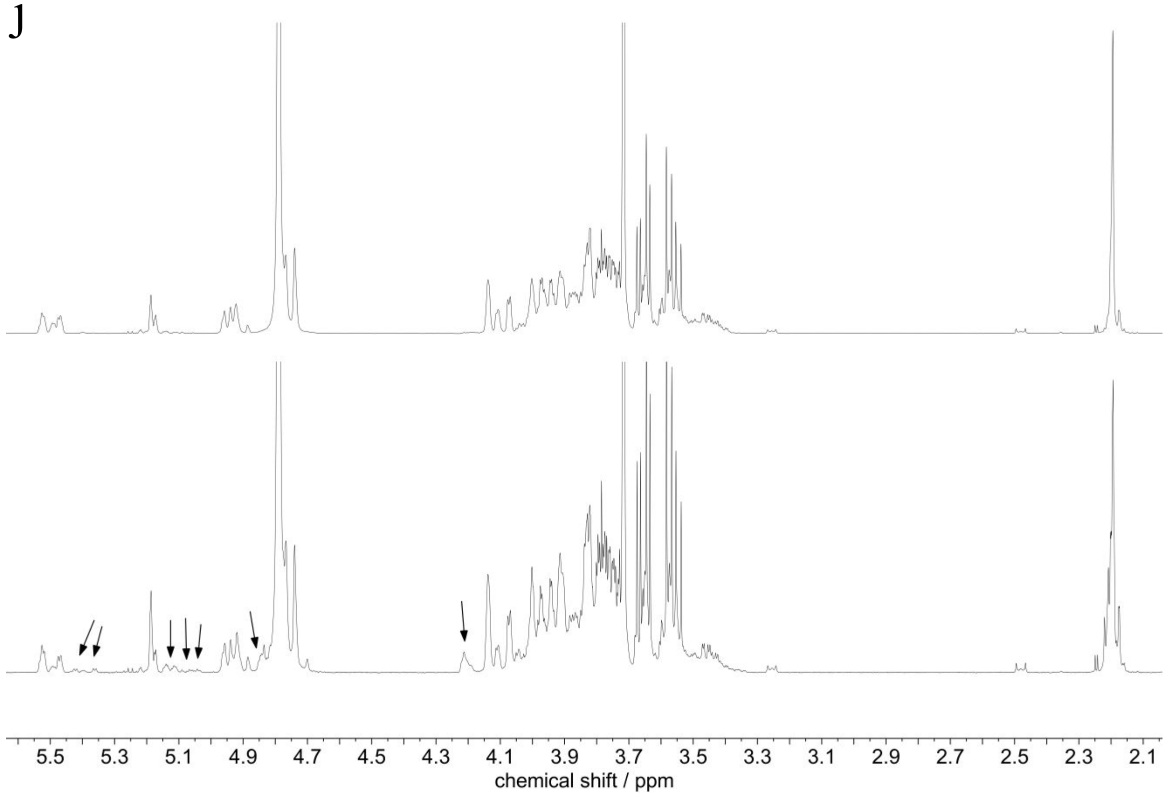
chemical shift / ppm

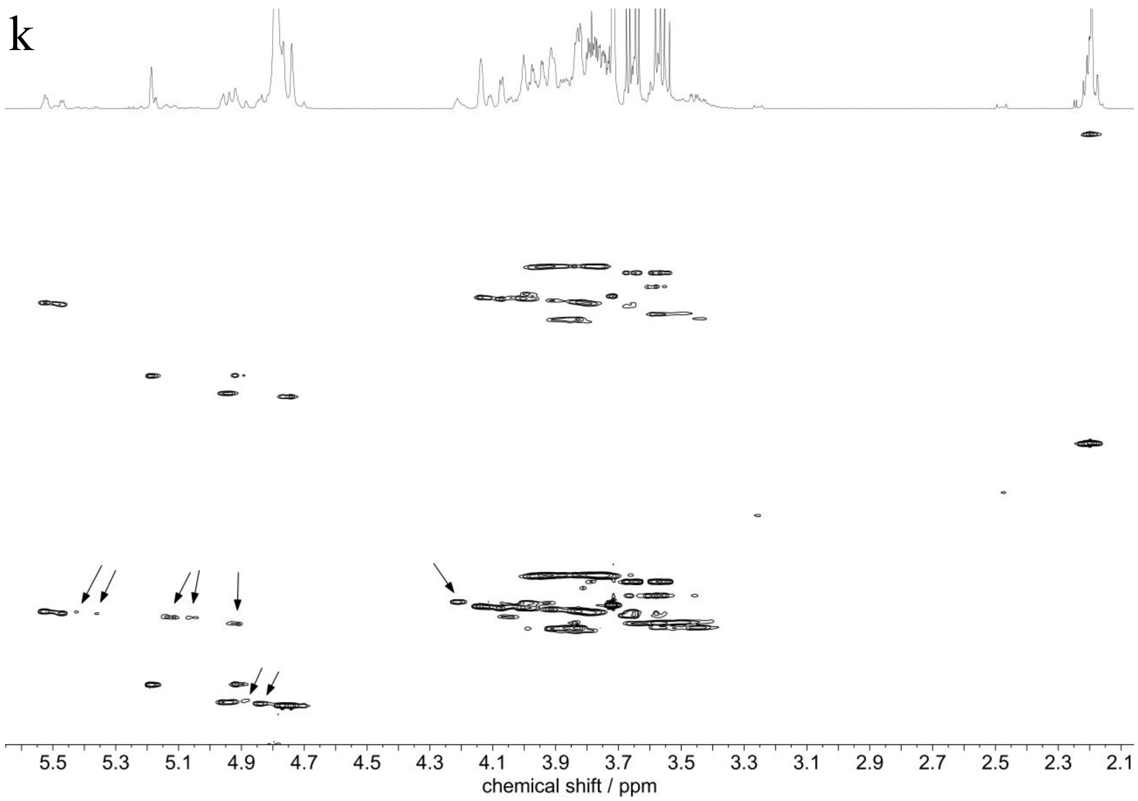


**Figure S4.** a) HMBC spectrum of acetylated mannotetraose products in $D_2O$. b) $^{1}H$-$^{1}H$ COSY spectrum of acetylated mannotetraose products in $D_2O$. c) HSQC spectrum of acetylated mannotetraose products in $D_2O$. d) 2D-selective HSQC spectrum of acetylated mannotetraose products in $D_2O$. e) 2D-selective HSQC spectrum of acetylated mannotetraose products in $D_2O$. f) TOCSY spectrum of acetylated mannotetraose products in $D_2O$. g) $^{1}H$-NMR spectrum (400 MHz, $D_2O$) of acetylated mannotetraose products. h) DEPTQ spectrum (100 MHz, $D_2O$) of acetylated mannotetraose products. i) HSQC-TOCSY spectrum of acetylated mannotetraose products in $D_2O$ (full spectrum of the partial spectrum shown in Figure 4). Analyses for Acetyl Migration j) and k). j) $^{1}H$-NMR spectrum (400 MHz, $D_2O$) of transacetylated mannotetraose before (upper spectrum) and after (lower spectrum) acetyl migration. New signals are marked by arrows. k) HSQC spectrum before (top) and after (bottom) acetyl migration in $D_2O$. New signals are marked by arrows.

## NMR Data of Mannotetraose

**Table S3.** Peak assignments for mannotetraose.

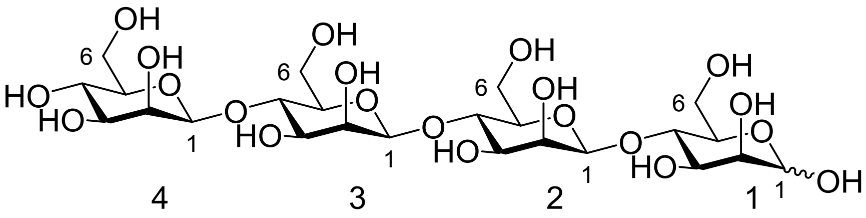


| | Mannose-unit | | | | |
|---|---|---|---|---|---|
| | 4 | 3 | 2 | 1-β | 1-α |
| 1 H<br>C | 4.77, s<br>103.2 | 4.74, s<br>103.1 | 4.74, s<br>103.1 | 4.92, d, $J^3$ = 0.7 Hz<br>96.7 | 5.19, d, $J^3$ = 1.3 Hz<br>96.8 |
| 2 H<br>C | 4.07, d, $J^3$ = 3.2 Hz<br>73.5 | 4.14<br>73.0 | 4.14<br>72.9 | 4.01<br>73.6 | 3.99<br>73.2 |
| 3 H<br>C | 3.66, dd, $J^3$ = 9.6, 3.2 Hz<br>75.7 | 3.84<br>74.4 | 3.84<br>74.4 | 3.84<br>74.6 | 3.92<br>73.8 |
| 4 H | 3.57 | 3.85 | 3.85 | 3.85 | 3.91 |
| C | 69.7 | 79.5 (4 peaks) | | | 79.7 |
| 5 H<br>C | 3.45, ddd, $J^3$ = 3.2 Hz 9.5<br>6.8 2.2 Hz<br>79.4 | 3.58<br>78.0 | 3.58<br>78.0 | 3.51<br>77.8 | 3.58<br>71.9 |
| 6 $H_1$<br>$H_2$<br>C | 3.74<br>3.96<br>64.0 | 3.79<br>3.92<br>63.5 | 3.79<br>3.92<br>63.5 | 3.79<br>3.92<br>63.5 | 3.78<br>3.86<br>63.5 |

a

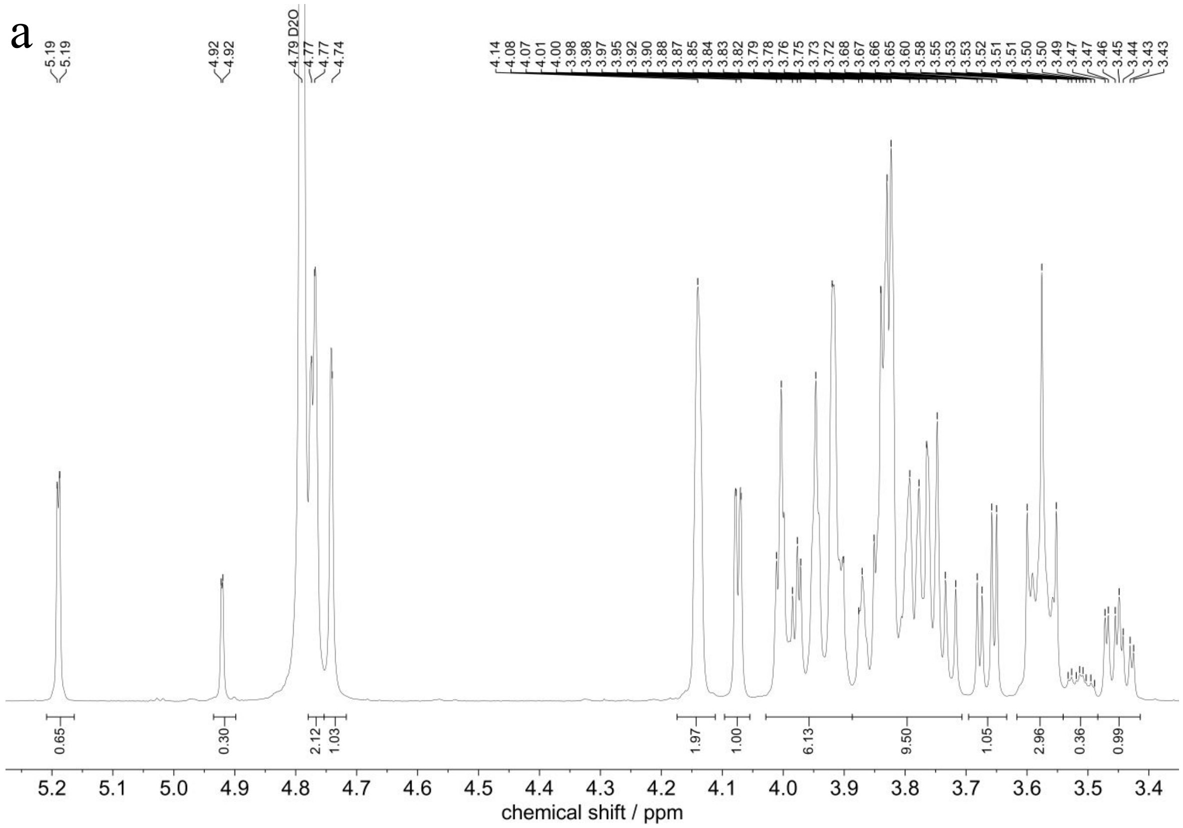


b

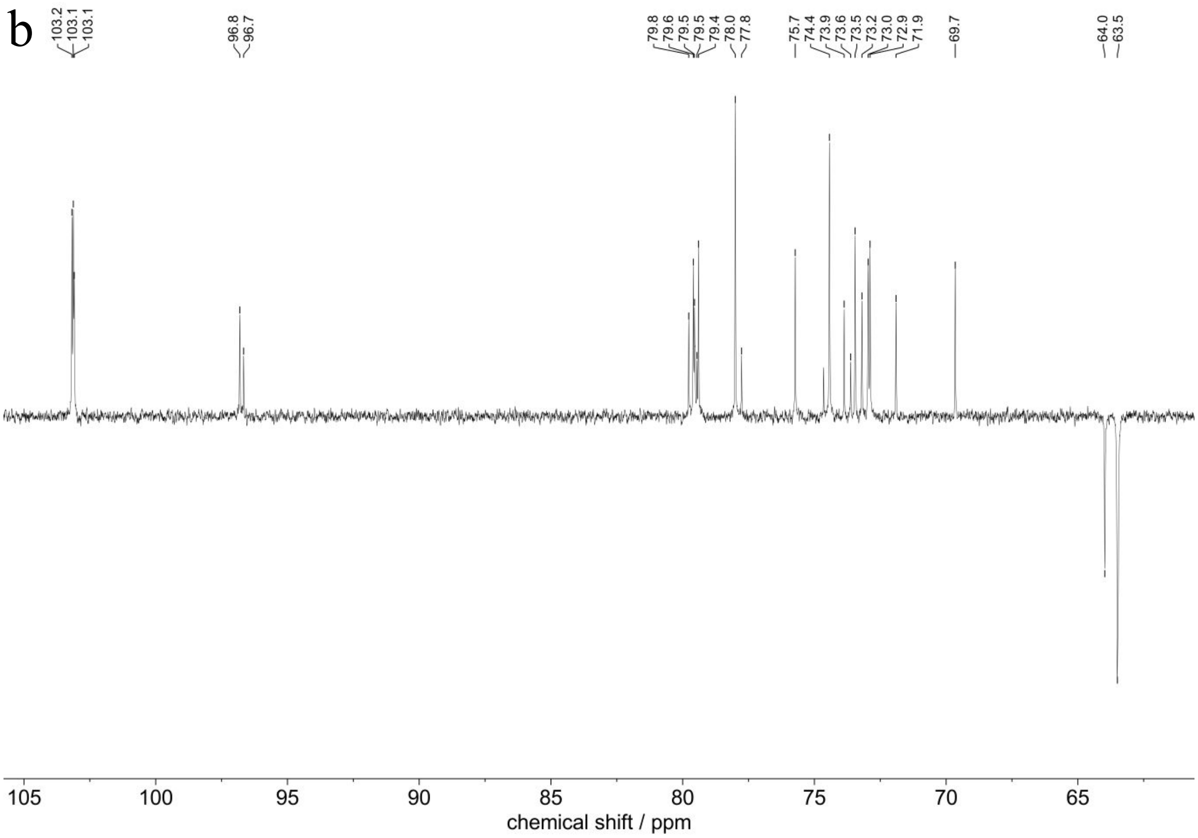

c

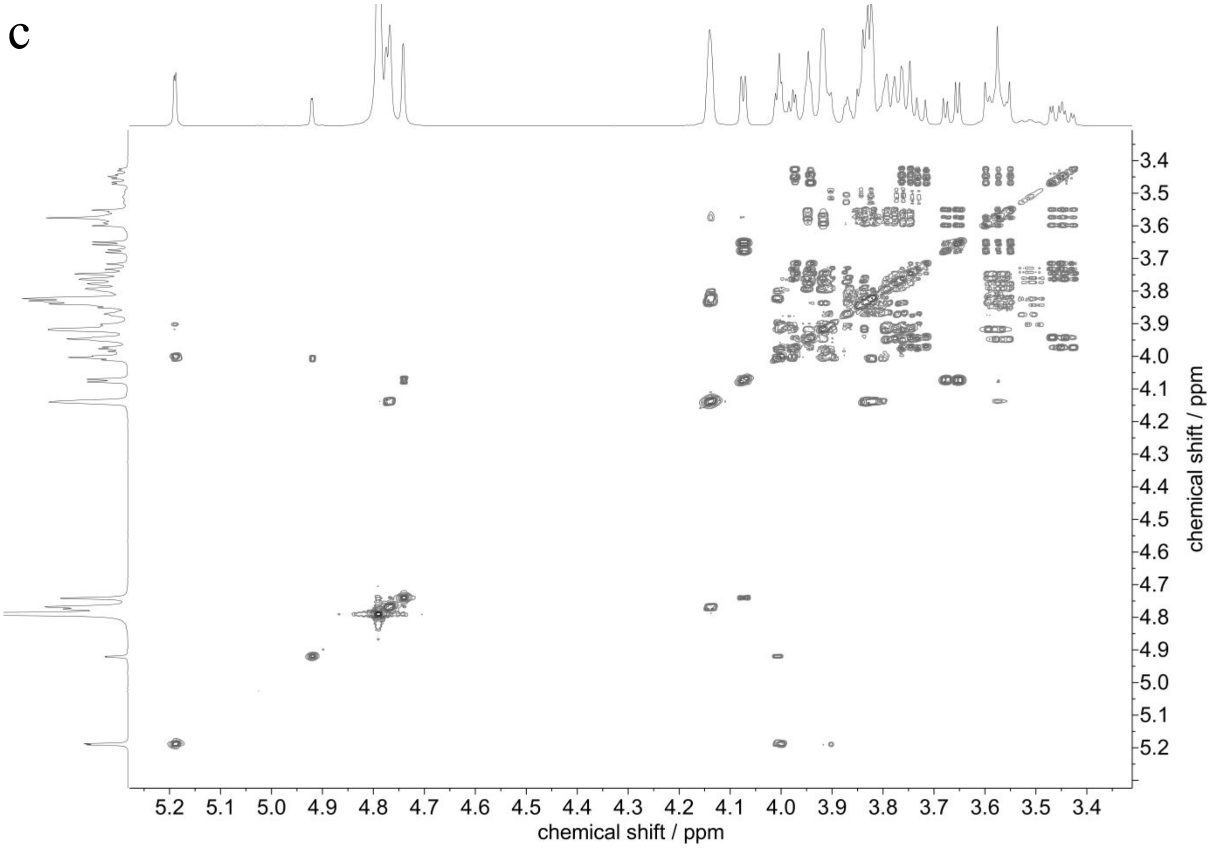
chemical shift / ppm
chemical shift / ppm

d

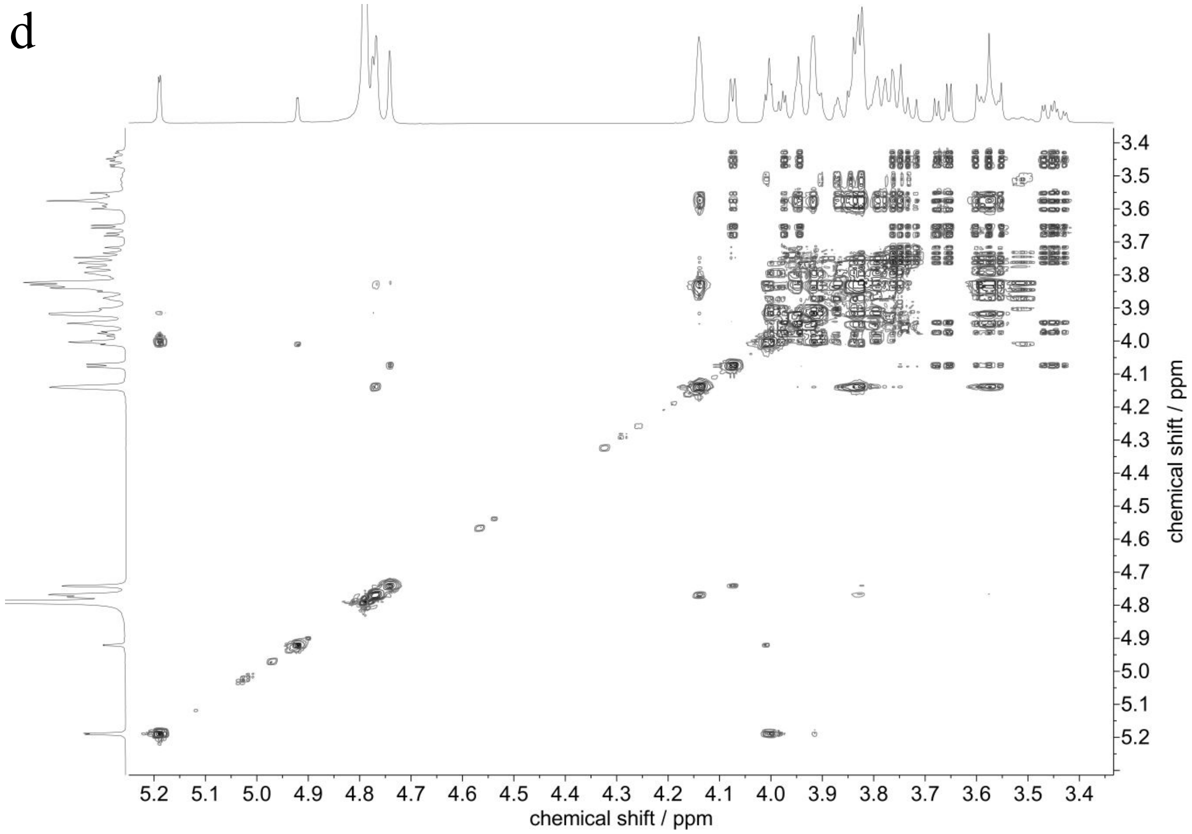
chemical shift / ppm
chemical shift / ppm

e

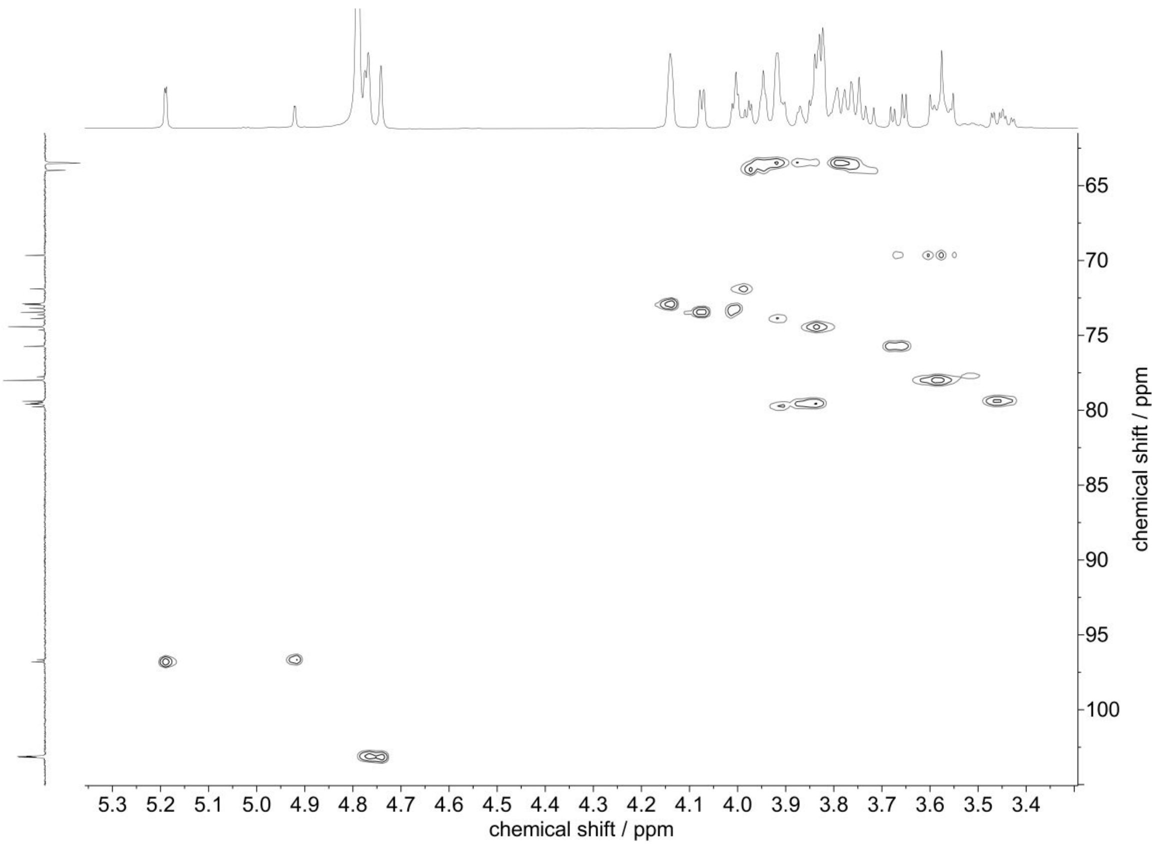

5.3
5.2
5.1
5.0
4.9
4.8
4.7
4.6
4.5
4.4
4.3
4.2
4.1
4.0
3.9
3.8
3.7
3.6
3.5
3.4
chemical shift / ppm
65
70
75
80
85
90
95
100
chemical shift / ppm


f

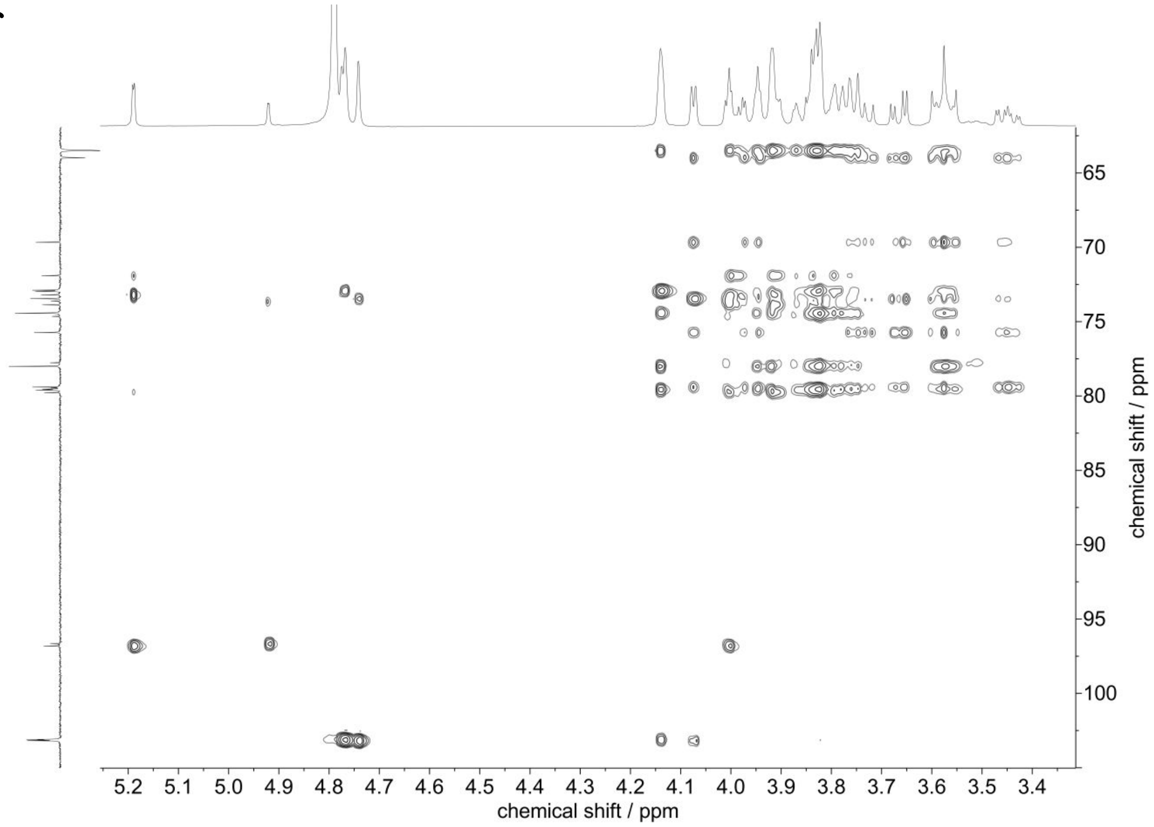

5.2
5.1
5.0
4.9
4.8
4.7
4.6
4.5
4.4
4.3
4.2
4.1
4.0
3.9
3.8
3.7
3.6
3.5
3.4
chemical shift / ppm
65
70
75
80
85
90
95
100
chemical shift / ppm

g

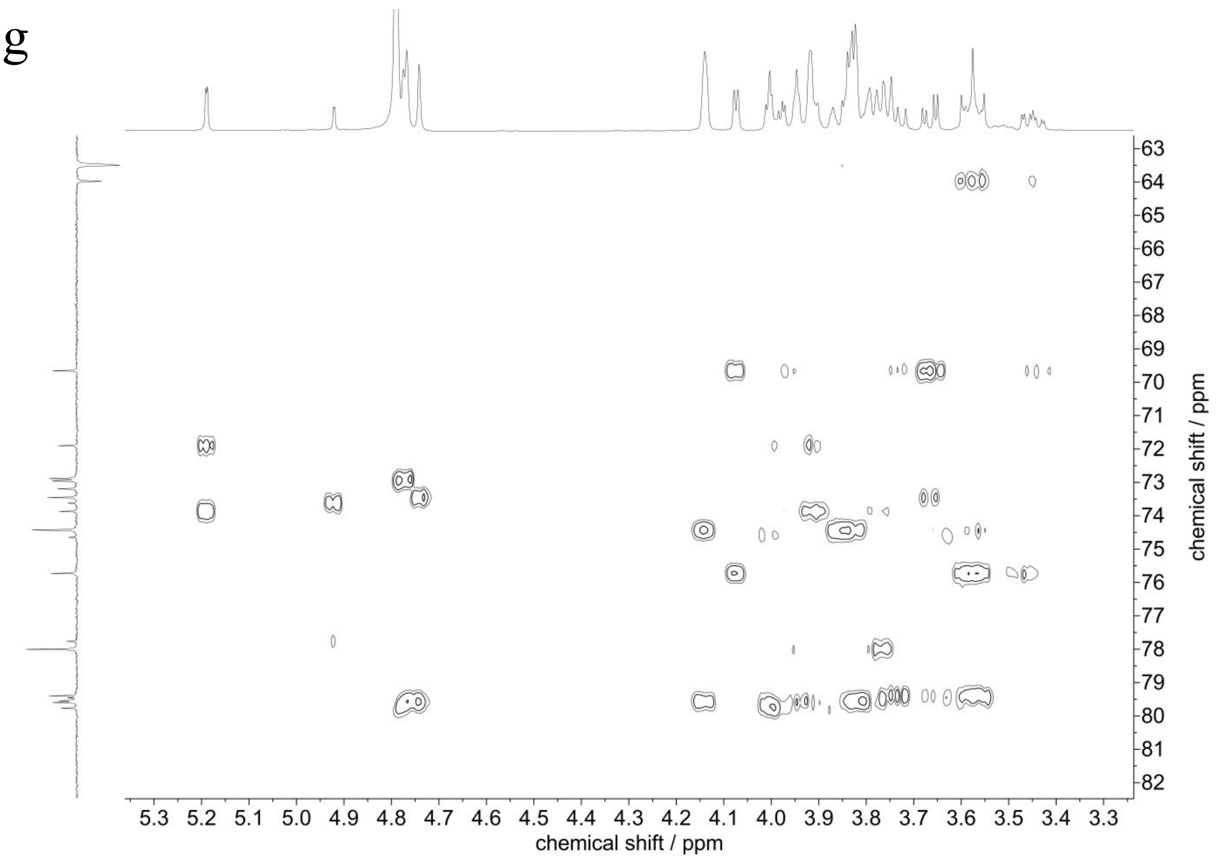


**Figure S5** a) $^{1}$H-NMR spectrum (400 MHz, $D_2O$) of mannotetraose. b) DEPTQ spectrum (100 MHz, $D_2O$) of mannotetraose. c) $^{1}$H-$^{1}$H COSY spectrum of mannotetraose in $D_2O$. d) TOCSY spectrum of mannotetraose in $D_2O$. e) HSQC spectrum of mannotetraose in $D_2O$. f) HSQC-TOCSY spectrum of mannotetraose in $D_2O$. g) HMBC spectrum of mannotetraose in $D_2O$.

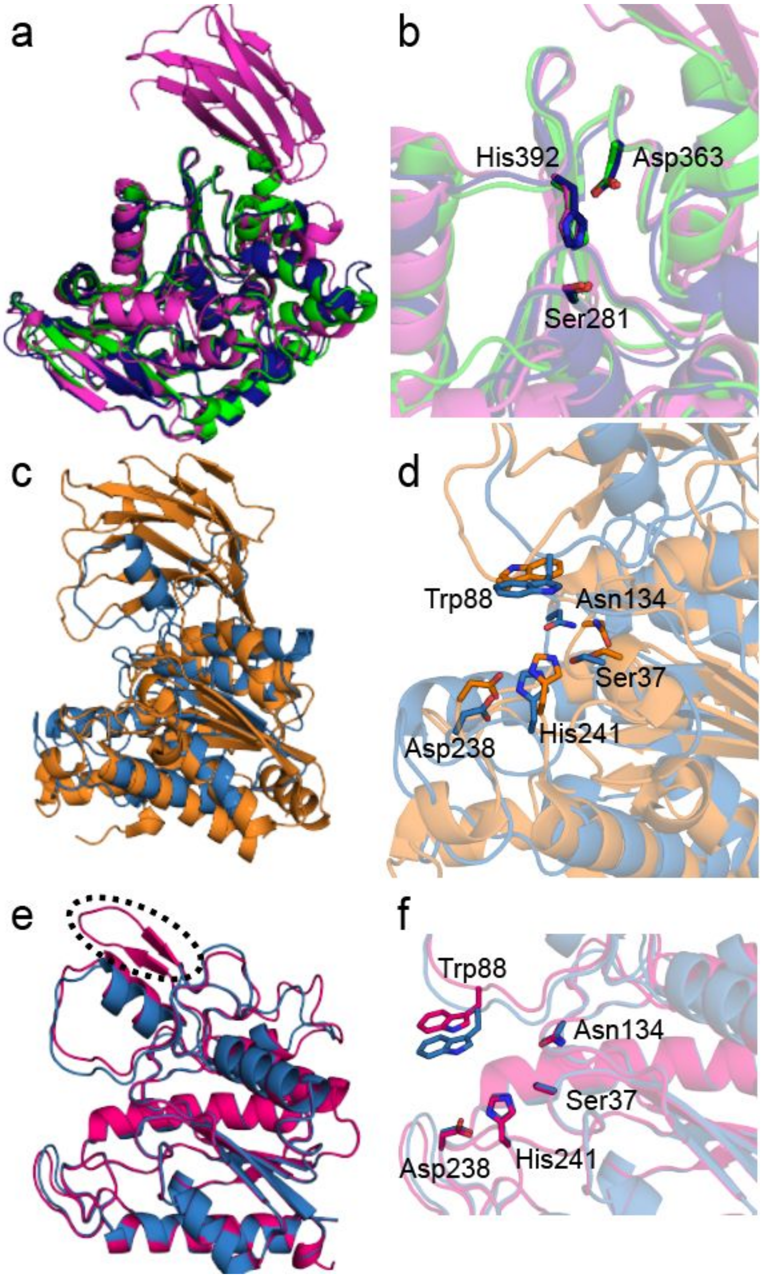
a
b
His392
Asp363
Ser281
c
d
Trp88
Asn134
Ser37
Asp238
His241
e
f
Trp88
Asn134
Ser37
Asp238
His241

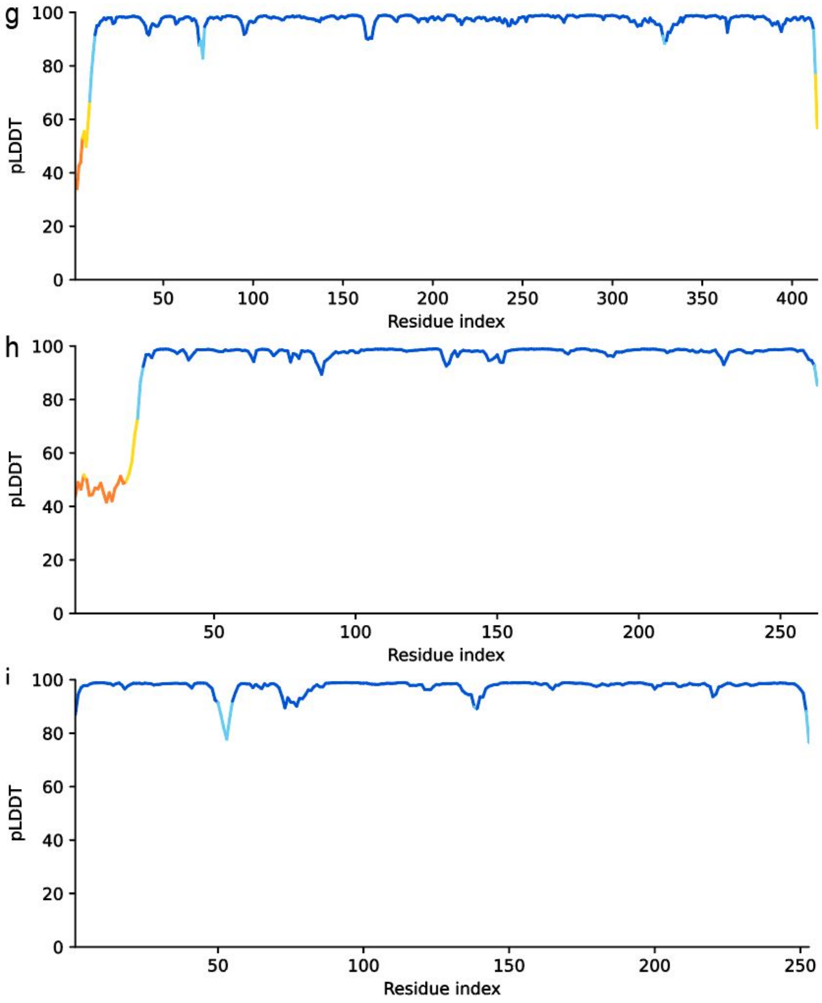


**Figure S6.** Predicted AlphaFold structures of *Bc*CE7 and *Bc*CE25. a) *Bc*CE7 (magenta) superimposed with CE7's from *Bacillus subtilis* (green) (PDB: 1ODS, RMSD = 1.27 Å) and *Paenibacillus* sp. R4 (dark blue) (PDB: 6AGQ, RMSD = 1.27 Å). b) The active site of all three CE7s with the catalytic triad Ser-His-Asp (*Bc*CE7 is numbered). c) *Bc*CE25 (blue) superimposed with *Ri*CE17 (orange) (PDB: 6HFZ) with an RMSD of 2.61 Å. d) Active site of *Bc*CE25 and *Ri*CE17. The predicted Ser-His-Asp catalytic triad of *Bc*CE25 (numbered), including the Trp for substrate stacking and Asn in the oxyanion hole, with the corresponding amino acids in *Ri*CE17 (Ser41-His193-Asp190, Trp326, and Asn110, respectively). e) *Bc*CE25 (blue) superimposed with a CE25 (pink) from a Bacillota member (Oscillospiraceae bacterium) (RMSD = 0.44 Å). The main structural difference is marked with a dotted circle. f) The catalytic site of CE25 from Bacteroidetes and Bacillota. pLDDT plots for the AlphaFold models g) *Bc*CE7, h) *Bc*CE25, and i) a CE25 representative from a Bacillota member (Oscillospiraceae bacterium). RMSD values are based on $C_\alpha$ atoms.

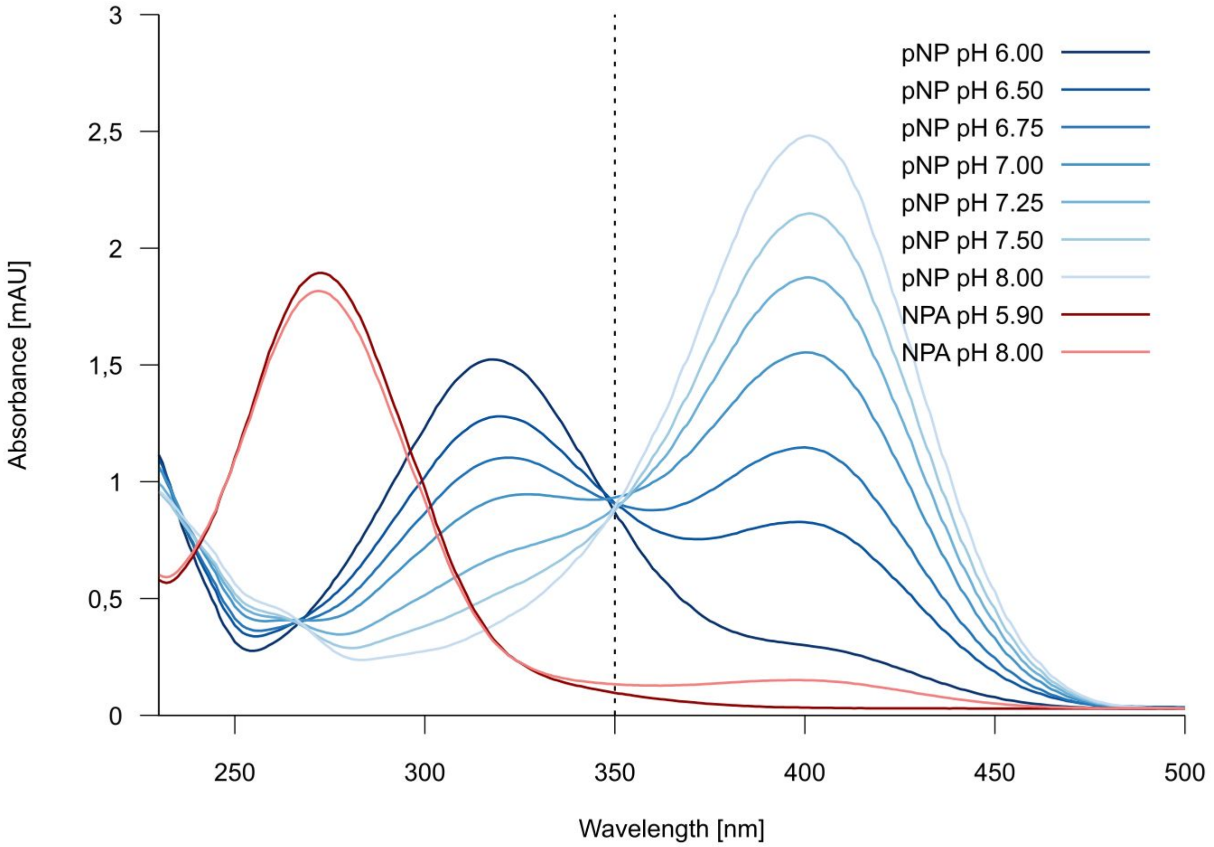


**Figure S7.** UV–Vis absorption spectra of *p*-nitrophenol (pNP) and *p*-nitrophenyl acetate (NPA) recorded under different pH conditions. Spectra of pNP were measured at varying pH values in assay buffer (100 mM sodium phosphate). For comparison, absorption spectra of NPA were recorded in 20 mM Tris buffer at pH 8.0 and 20 mM sodium phosphate with 200 mM NaCl at pH 5.9. The dashed line indicates 350%nm, the wavelength used to monitor enzyme activity in assays.

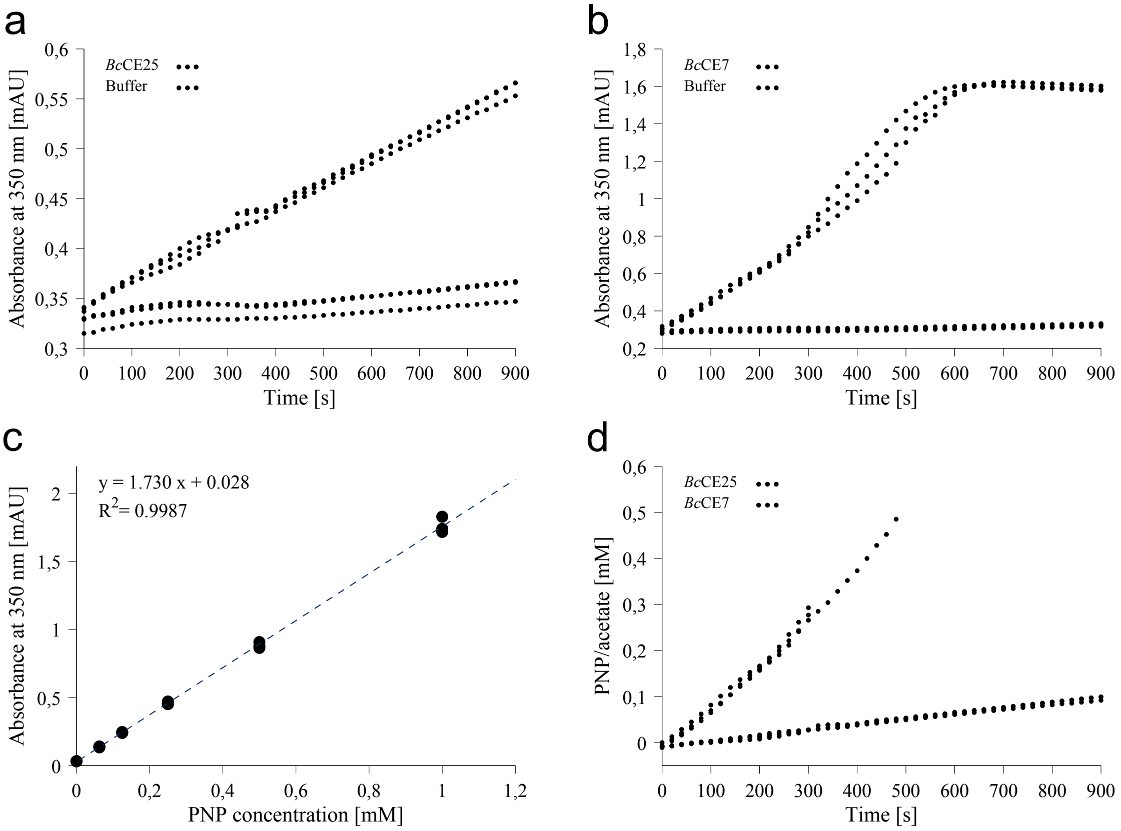


**Figure S8.** Activity assays with pNP-acetate. (a,b) Raw absorbance traces recorded at 350%nm for reactions catalyzed by 100 nM BcCE25 (a) and 50 nM BcCE7 (b) in 100 mM phosphate buffer at pH 7.25 and 37 ˚C alongside buffer controls. Three independent replicates are shown for each enzyme. (c) pNP standard curve obtained under the same buffer conditions and wavelength, used to convert absorbance values to product concentration. (d) Processed reaction progress curves for both enzymes after buffer subtraction and conversion to pNP/acetate concentrations using the standard curve shown in (c). Only the initial linear range of the reaction is displayed and was used for activity determination.

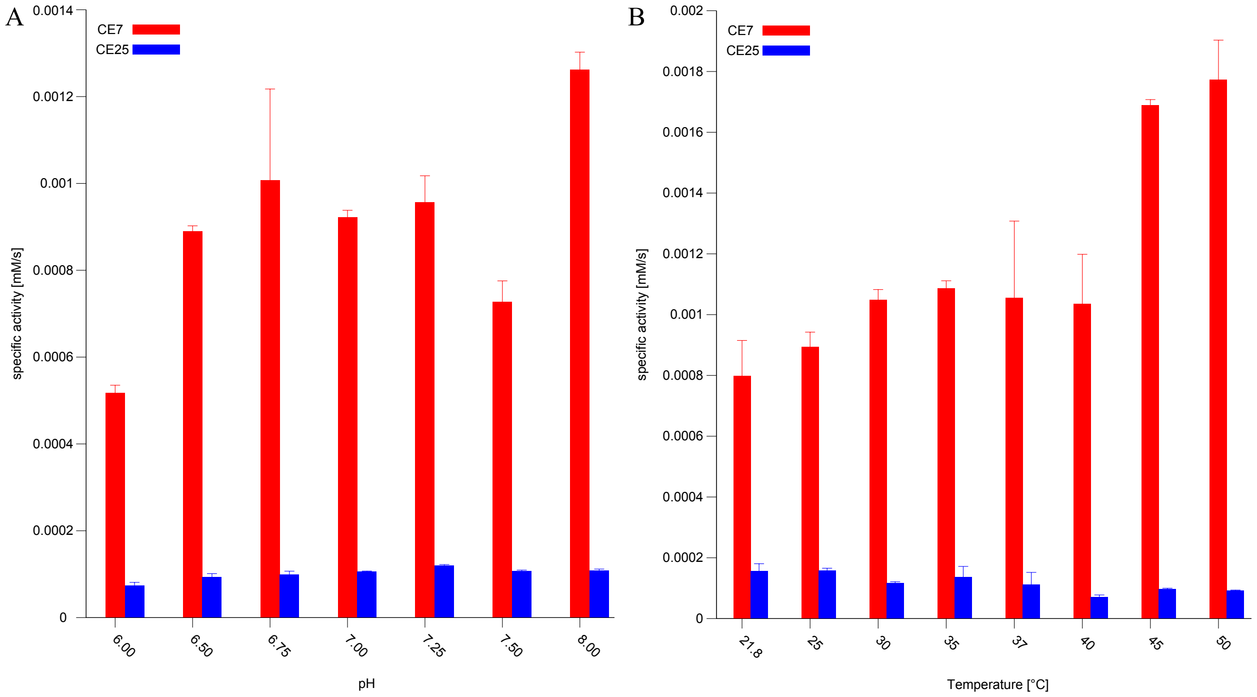


**Figure S9.** pNP-acetate hydrolysis by *Bc*CE7 and *Bc*CE25 in different pH (a) and different temperature (b). The reactions were performed with 50 nM *Bc*CE7 and 100 nM *Bc*CE25 with 1 mM pNP-acetate in 100 mM sodium phosphate buffer at varying pH and 37 ˚C (a) or in 100 mM sodium phosphate buffer pH 7.25 and varying temperature (b).

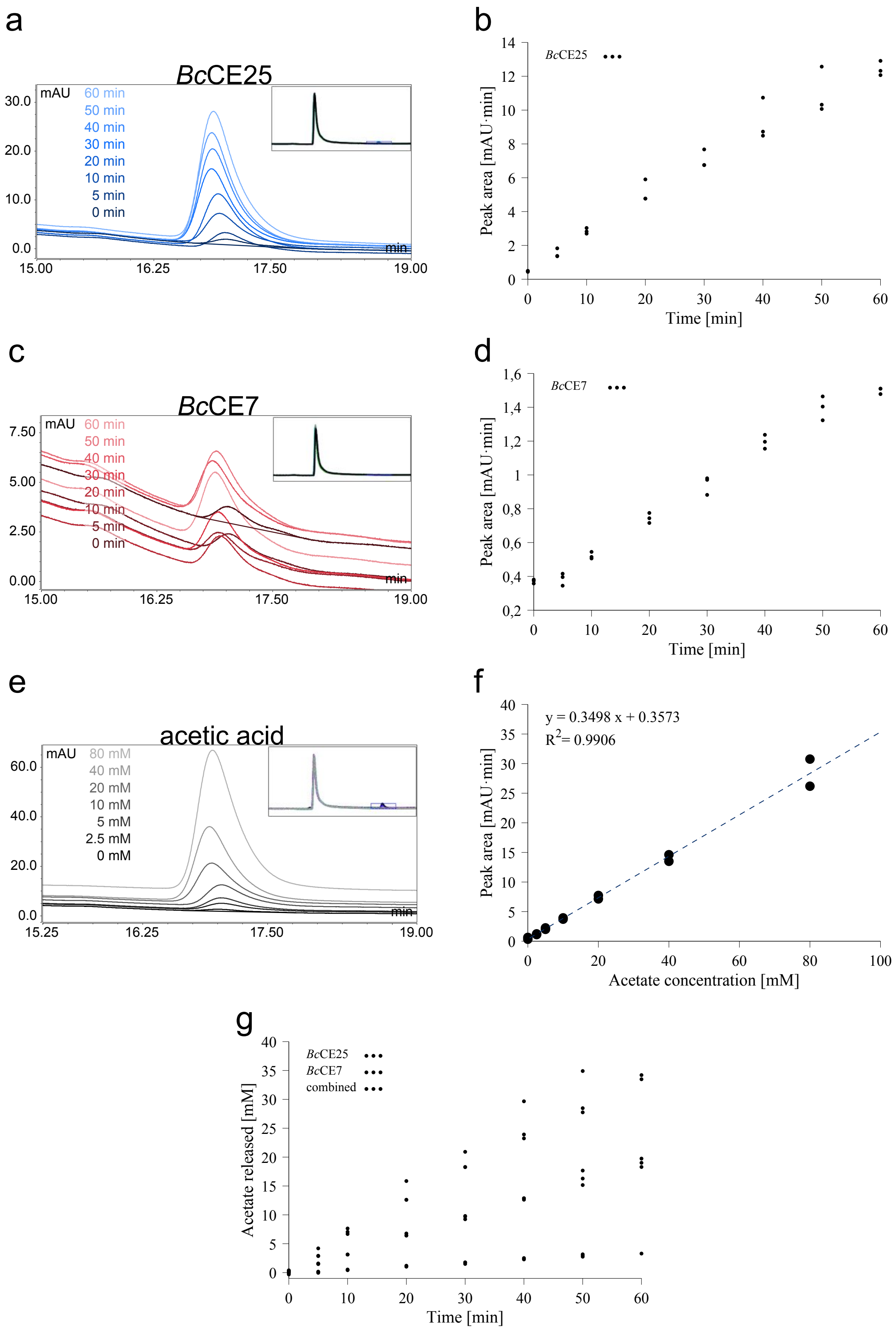
a
BcCE25
mAU
60 min
50 min
40 min
30 min
20 min
10 min
5 min
0 min
min
b
BcCE25
Peak area [mAU·min]
Time [min]
c
BcCE7
d
BcCE7
e
acetic acid
80 mM
40 mM
20 mM
10 mM
5 mM
2.5 mM
0 mM
f
y = 0.3498 x + 0.3573
R2= 0.9906
Acetate concentration [mM]
g
BcCE25
BcCE7
combined
Acetate released [mM]

**Figure S10.** Acetate release assay with AcGGM. (a,c,e) Representative Rezex HPLC chromatograms of reaction samples (a,c) and acetate standards (e), zoomed-in on the acetate elution peak. Inlets display the corresponding full chromatograms. (b,d) Time-dependent acetate peak areas obtained from HPLC analysis of reactions catalyzed by 50 nM enzymes *Bc*CE25 (b) and *Bc*CE7 (d) in 100 mM sodium phosphate at pH 7.25 and 37 ˚C. Data from three independent replicate reactions are shown. (f) Acetate standard curve generated in the presence of AcGGM substrate background, showing two technical replicates, and used for conversion of peak areas to acetate concentration. (g) Reaction progress curves showing acetate release over time after conversion of peak areas using the standard curve in (f). Data are shown for *Bc*CE25 alone, *Bc*CE7 alone, and a mixture of 25 nM of both enzymes. Only the initial linear range of the reaction is displayed and was used for activity determination.

**Table S4.** Specific activity and apparent turnover rate by *Ri*CE2 and *Ri*CE17 on *Ri*GH26-digested spruce AcGGM. The parameters were determined based on triplicate measurements of the amount of acetate released linearly within the first hour. All reactions were run with 50 nM enzyme concentration in phosphate buffer at pH 7.25 and 37 ˚C.

| Deacetylation of Norway spruce AcGGM | Specific activity [µmol · $min^{-1}$ · $mg^{-1}$] | Apparent turnover rate [$s^{-1}$] |
|---|---|---|
| *Ri*CE2 | 62.9 (± 4.3) | 42.4 (± 2.8) |
| *Ri*CE17 | 54.2 (± 5.1) | 38.3 (± 3.6) |
| *Ri*CE2 + *Ri*CE17 | | 51.8 (± 2.4) |
